# Preparation of Whole-mount Mouse Islets on Vascular Extracellular Matrix for Live Islet Cell Microscopy

**DOI:** 10.21769/BioProtoc.4868

**Published:** 2023-11-05

**Authors:** Kung-Hsien Ho, Guoqiang Gu, Irina Kaverina

**Affiliations:** 1 Department of Cell and Developmental Biology, Vanderbilt University, Nashville, TN, USA; 2 Program in Developmental Biology and Center for Stem Cell Biology, Vanderbilt University, Nashville, TN, USA

**Keywords:** Pancreatic β cells, Islet attachment, Islet ex vivo culture, Islet flattening, Human ECM, Live islet cells visualization

## Abstract

Pancreatic islet β cells preferentially secrete insulin toward the plasma membrane, making contact with the capillary extracellular matrix (ECM). Isolated islets separated from the exocrine acinar cells are the best system for cell biology studies of primary β cells, whereas isolated islets lose their capillary network during ex vivo culture. Providing the appropriate extracellular signaling by attaching islets to vascular ECM-coated surfaces can restore the polarized insulin secretion toward the ECM. The guided secretion toward ECM-coated glass coverslips provides a good model for recording insulin secretion in real time to study its regulation. Additionally, β cells attached to the ECM-coated coverslips are suitable for confocal live imaging of subcellular components including adhesion molecules, cytoskeleton, and ion channels. This procedure is also compatible for total internal reflection fluorescence (TIRF) microscopy, which provides optimal signal-to-noise ratio and high spatial precision of structures close to the plasma membrane. In this article, we describe the optimized protocol for vascular ECM-coating of glass coverslips and the process of attachment of isolated mouse islets on the coverslip. This preparation is compatible with any high-resolution microscopy of live primary β cells.

Key features

• Optimized coating procedure to attach isolated islets, compatible for both confocal and TIRF microscopy.

• The ECM-coated glass coverslip functions as the artificial capillary surface to guide secretion toward the coated surface for optimal imaging of secretion events.

• Shows the process of islets attachment to the ECM-coated surface in a 6-day ex vivo culture.

**Graphical overview**


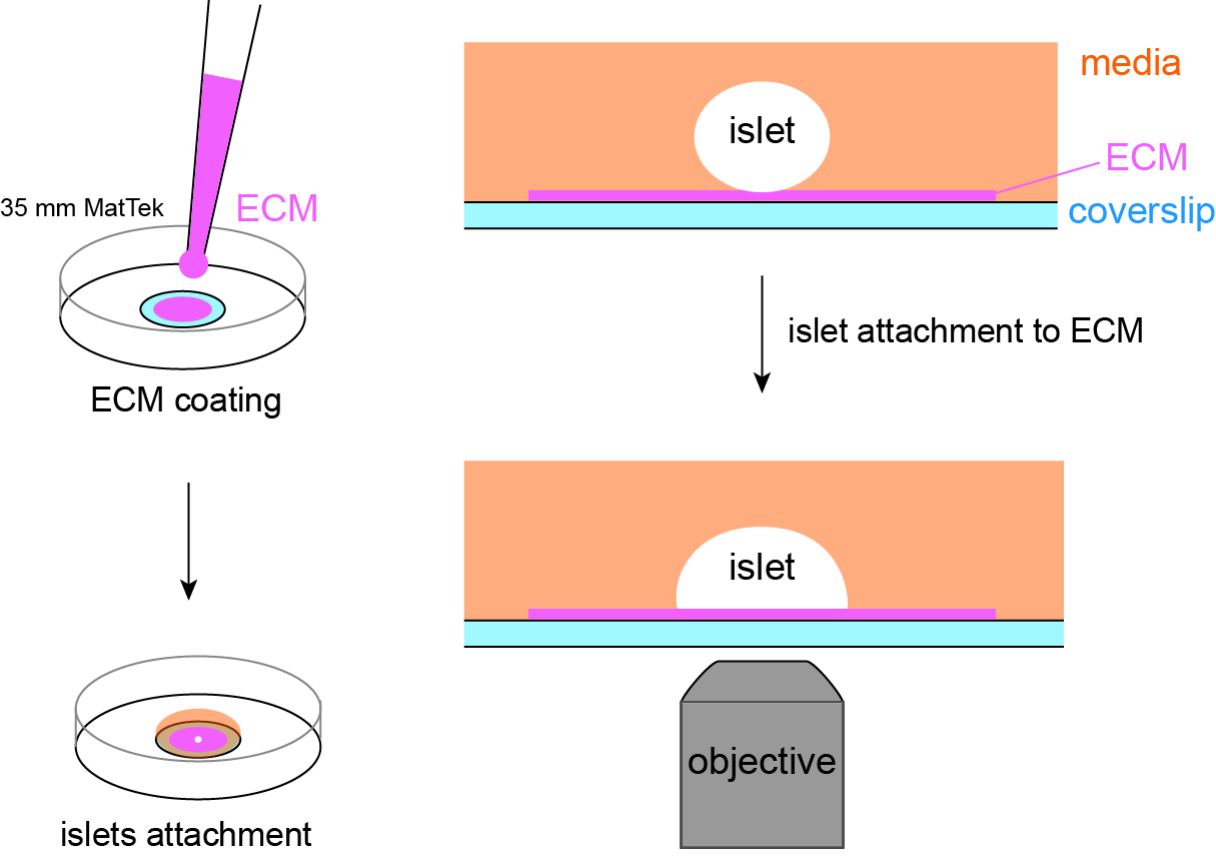


## Background

Pancreatic β cells regulate blood glucose homeostasis by secreting insulin to the blood stream in response to elevated glucose levels. The secretion of insulin is tightly regulated to prevent over-secretion, which causes hypoglycemia, or under-secretion, which in the long term leads to diabetes. Islet β cells preferentially secrete insulin toward the plasma membrane, making contact with the capillary extracellular matrix (ECM) or basement membrane (Low et al., 2014; Gan et al., 2018). Isolated islets lose their capillaries and the polarity of β cells during ex vivo culture. When studying β-cells secretion, recording secretion events on the plasma membrane making contact with the vascular ECM can better recapitulate the polarized insulin secretion of β cells in vivo. Coating coverslips with human ECM, derived from placenta and containing the main components of vascular ECM, is an ideal surface for islet attachment to study insulin secretion (Patterson et al., 2000; Arous and Wehrle-Haller, 2017; Barillaro et al., 2022). The coated coverslip functions as the artificial capillary surface to guide the secretion of attached islet β cells toward it and is suitable for high-resolution imaging to record secretion events.

Total internal reflection fluorescence (TIRF) microscopy is a powerful tool to study subcellular structures in attached β cells, such as adhesion molecules, cortical actin cytoskeleton, ion channels, and lipids in the plasma membrane. Another powerful application of TIRF microscopy is to record insulin secretion from individual β cells in real time. Multiple studies have reported different approaches to labeling insulin vesicles (Ma et al., 2004; Ivanova et al., 2013; Schifferer et al., 2017). Integration of an engineered insulin fusion probe to the genome of β cells in isolated rodent or human islets is usually technically challenging. Label-free approaches bypass this challenge and the possibility that a fluorescence probe may interfere with the maturation or secretion of insulin (Pouli et al., 1998; Schifferer et al., 2017). Successful label-free recording of insulin exocytosis has been reported using either two-photon microscopy or correlative scanning ion conductance microscopy-fluorescence confocal microscopy (Takahashi et al., 2002; Bednarska et al., 2021). Our lab has previously established a novel label-free approach to record insulin secretion using TIRF microscopy and a cell-impermeable zinc-sensitive fluorescence dye, FluoZin^TM^-3 (Gee et al., 2002; Zhu et al., 2015). The fusion of individual insulin vesicles to the plasma membrane transiently elevates local zinc concentration, which activates FluoZin^TM^-3 to emit fluorescence upon excitation. This strategy allows visualization of insulin secretion at the cell–ECM interface with high spatial precision in real time. The conventional coating procedure for islet attachment and confocal microscopy includes a layer of matrigel between the coverslip and the ECM-coating. The matrigel layer provides a three-dimensional-like cushion to facilitate islet attachment but is not compatible with TIRF microscopy due to its thickness. In this article, we describe the details of an optimized ECM-coating and attachment of isolated islets on glass coverslips, which is compatible with TIRF microscopy and any high-resolution live-cell microscopy.

## Materials and reagents

1. Mice [*GFP-Lifeact; Ins2^Apple^*, 12–20 weeks (Riedl et al., 2008; Stancill et al., 2019)]
2. Human extracellular matrix (ECM) (Corning, catalog number: 354237)
3. Fetal bovine serum (FBS) (Atlanta Biologicals, catalog number: S11550)
4. RPMI 1640 (Gibco, catalog number: 11875)
5. Penicillin-Streptomycin solution (Pen-Strep) (Gibco, catalog number: 15140)
6. Nalgene^TM^ Rapid-Flow^TM^ Sterile Disposable Filter Units, 0.2 μm PES membrane (Thermo Fisher Scientific, catalog number: 569-0020)
7. Hanks’ Balanced Salt Solution with calcium & magnesium (HBSS) (Gibco, catalog number: 21-020-CV)
8. Collagenase from *Clostridium histolyticum* (Millipore Sigma, catalog number: C5138, lot number: 0000164940)
9. Islet media (see Recipes)
10. Collagenase solution (see Recipes)
11. ECM coating solution (see Recipes)

## Recipes

1. **Islet media**
  RPMI-1640 (the media contains 11 mM glucose)
  10% heat-inactivated FBS
  100 U/mL penicillin-100 mg/mL streptomycin
  Filter the media through a 0.2 μm PES membrane, store at 4 °C, and used within one month of preparation.
2. **Collagenase solution**
  0.5 mg/mL collagenase in HBSS
  Store aliquots (1 mL) of the prepared solution at -20 °C for up to one year.
3. **ECM coating solution**
  9 μg/mL ECM in islet media
  Store aliquots (100 μL) of the prepared solution at -20 °C for up to two years.

## Equipment

1. Plasma cleaner (Harrick Plasma, model: PDC-001)
2. Nikon A1R laser scanning confocal microscope
3. CFI Apochromat TIRF 100×/1.45 oil objective (Nikon)
4. Olympus SZH10 dissection scope
5. MatTek glass bottom dishes, 35 mm dish, 10 mm microwell (MatTek, catalog number: P35G-1.5-10-C)
6. 150 mm dish (Corning, catalog number: 430599)
7. Tissue wipers (VWR, catalog number: 82003-820)
8. 5 mL syringe (BD, catalog number: 309646)
9. Needle [BD, catalog numbers: 305109 (27 G 1/2) and 305106 (30 G 1/2)]
10. 50 mL centrifuge tubes (VWR, catalog number: 525-1075)
11. Precision water bath (Precision Scientific, Model 182)
12. 60-mm polystyrene Petri dish (Thermo Fisher Scientific, catalog number: AS4052)
13. Water-jacked CO_2_ incubator (NuAire, model: NU-8700 series 5)

## Procedure

**Day 0**

1. The collagenase digestion of mouse pancreas and islet isolation follows the published procedure (Li et al., 2009; Ho et al., 2023). Briefly, mouse pancreata are injected with 2 mL of collagenase solution (see Recipes) using a 5 mL syringe and a 27 G 1/2 needle (or a 30 G 1/2 needle for smaller mice) through the common bile duct. The pancreata are digested in a 50 mL centrifuge tube in a water bath at 37 °C for 16 min with gentle shaking every 4 min. The digested homogenate is washed using 10 mL (per animal’s pancreata) of chilled islet media (see Recipes) with vigorous shaking and centrifuged at 700× *g* for 2 min at 4 °C. Islets are hand-picked using a P20 pipette with 200 μL tips to a 60 mm Petri dish with islet media under a dissection scope. Repeat the picking three times to separate islets from pancreatic acinar cells (Note 1). The isolated islets are incubated in a water-jacked incubator with 5% CO_2_ (Note 2).
2. Remove the lid and place the 35 mm MatTek dish with a 10 mm microwell in the plasma cleaner. Seal the chamber and establish vacuum. Set the RF (radio frequency) level to medium and initiate the cleaning for 1 min (Note 3). Place the lid back immediately after retrieving the dish from the cleaner. The plasma-cleaned MatTek dish can be stored in its packaging sleeve for six months at room temperature, but a coated dish is for immediate use.
3. Coat the coverslip of a plasma-cleaned MatTek dish by applying a small volume (~5 μL) of ECM coating solution (see Recipes) to the center of the coverslip (Figure 1A). When coating MatTek dishes, instead of measuring the exact volume of coating solution for each dish, we use a P100 pipette holding enough coating solution, push out a small amount of solution from the tip, smear it on the area to be coated, and suck excess solution back to the tip. This ensures the coating is not too thick to interfere with the downstream TIRF microscopy. We do not coat the entire coverslip to reduce the chance of an islet attaching to the edge of the microwell.
  **Figure 1.**
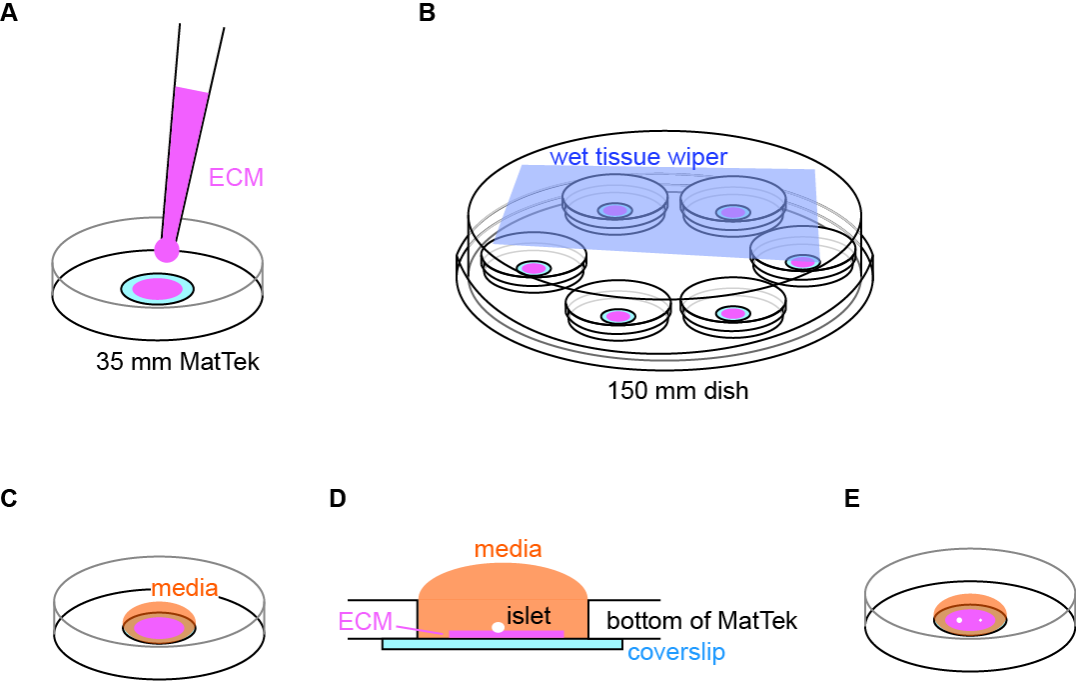
Illustration of coating and setup for islet attachment. (A) Schematic of coating of extracellular matrix (ECM) to the center of coverslip. (B) Schematic of MatTek dishes arrangement in a humidified 150 mm dish. (C) Schematic of media added and contained inside the microwell. (D) Side view of the MatTek dish showing the media contained inside the microwell. (E) Schematic showing two islets of different size plated per MatTek dish.
4. Cover the MatTek dish with its own lid and place it on the lid of a 150 mm dish. Create a humidified chamber by placing a sheet of tissue wiper wetted with distilled water on the bottom of the 150 mm dish and place the MatTek dish inside such that the tissue is at the top of the chamber (Figure 1B). Incubate at 37 °C in a water-jacked incubator for 10 min to allow ECM coating on the coverslip.
5. Wash the coated coverslip once by pipetting 100 μL of islet media to the microwell and aspirate it from its edge that is not coated with ECM. Refill the microwell with 100 μL of islet media (Figure 1C). During washing and refilling, the media should be contained inside the microwell. This helps to retain the surface tension of islet media and constrain islet attachment inside the microwell (Figure 1D). If the media covers the plastic dish bottom, the planted islets may float out of the microwell and attach to the plastic.
6. Under a dissection scope, transfer 1–2 islets to the center of the microwell using a P20 pipette (Figure 1E) (Note 4).
7. Place the MatTek dish in the 150 mm dish (the humidified chamber) and carefully move it to the incubator (Figure 1B). Choose islets smaller than 120 μm in diameter for better dye penetration to the space between the islet attachment surface and the coverslip in downstream imaging. Without a wet tissue wiper inside the 150 mm dish, the small volume of 100 μL of islet media will dry out overnight even in a water-jacketed incubator (Note 5).
8. Incubate it for two days. There is no need to replace media or take the MatTek dish out to check the status of islets before day 2 (see below). To follow the attachment process on the ECM-coated coverslip, islets isolated from the *GFP-Lifeact; Ins2^Apple^* mouse (Riedl et al., 2008; Stancill et al., 2019) are shown here. GFP-Lifeact labels cortical actin to delineate cell borders and *Ins2^Apple^ (Ins2* promoter driven H2B-mApple) marks β cells (Figure 2A). Approximately 70% of islets loosely attach to the ECM overnight (Figure 2B–2C). Taking the MatTek dish incubated overnight out for observation can easily stir up those loosely attached islets. Most islets (~90%) are firmly attached to the ECM after two nights.
  **Figure 2.**
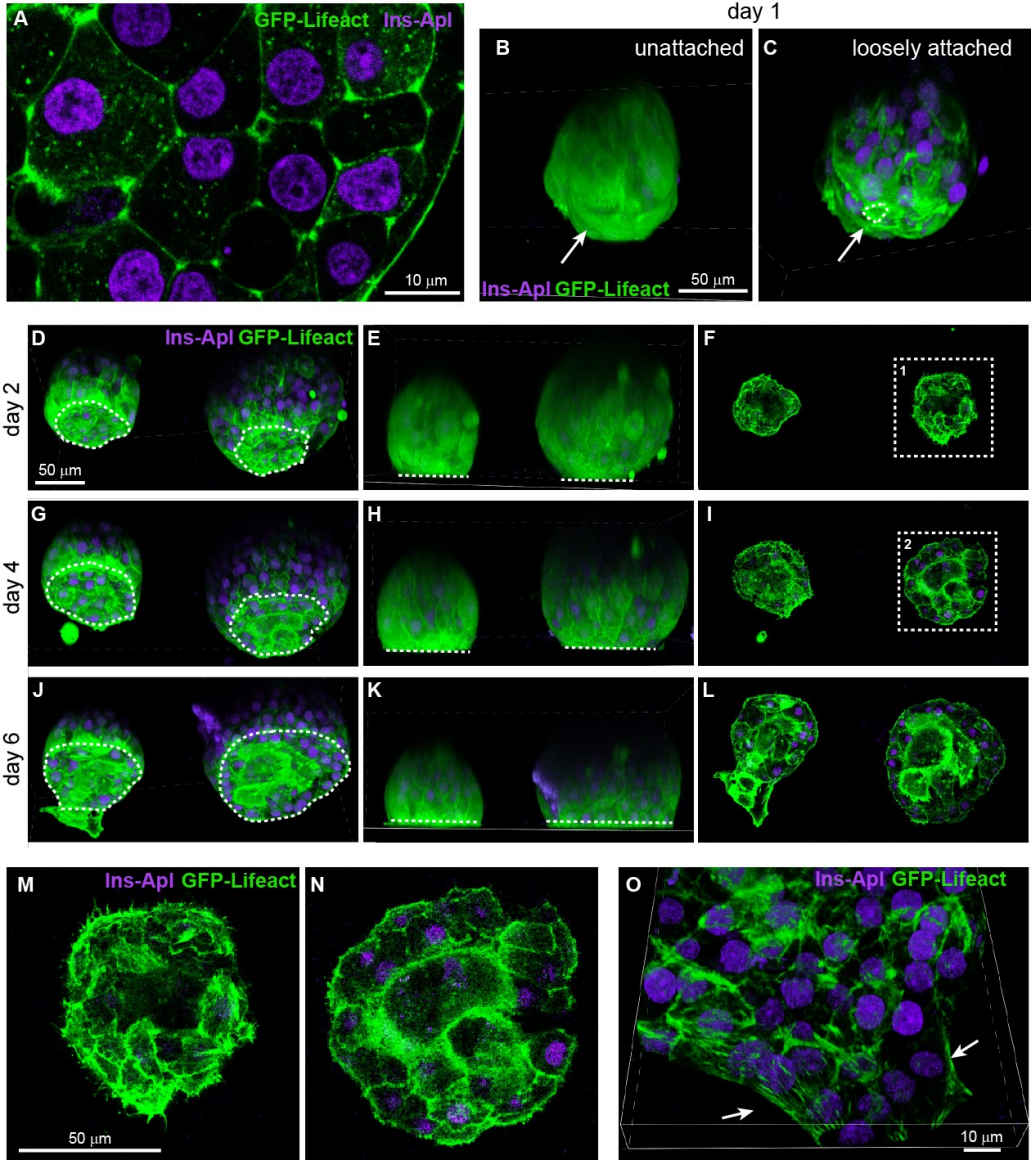
Attachment and flattening process of isolated mouse islets. (A) Single slice of confocal microscopy image of an islet isolated from the *GFP-Lifeact; Ins2^Apple^* mouse. GFP-Lifeact (green) delineates cell borders and β-cells nuclei are labeled by *Ins2^Apple^* (magenta). (B–C) Projected images of isolated islets incubated on extracellular matrix (ECM)-coated coverslip for one day. Dashed line delineates the attachment site of an islet. Arrow indicates the side in contact with the coverslip. (D–N) Confocal images of isolated islets incubated on ECM-coated coverslip for 2–6 days. D, G, and J show projected images. E, H, and K show the side view of the projected images. F, I, and L show single slice of confocal images at the z-plane of attachment. M and N show the enlargement of image at dashed square 1 and 2 respectively. Dashed lines delineate the attachment sites of islets (D, G, and J) or represent the location of coverslips (E, H, and K). (O) Projected image of an isolated islet attached to ECM-coated coverslip and flattened to single-cell-layer thickness at the periphery of the islet. Arrows indicate the periphery of the flattened islet.

**Day 2**

1. Take the dish out from the incubator and gently add 150 μL of islet media from the edge of the microwell under a dissection scope. Because the majority of islets have now attached to the ECM on the coverslip, they are resistant to minor vibrations while moving the dish and it is okay to let the media exceed the microwell (Figure 2D–2F and 2M). Do not remove old media before adding the 150 μL of fresh media. If the media is removed and then replaced, attached islets will be lifted up above the surface of media. For this reason, there should be no media change during the entire ex vivo culture of attached islets. Instead, we add more fresh media to the dish every other day.
2. Incubate the attached islets for two more days.

**Day 4**

Gently add 500 μL of islet media from the edge of the microwell. The media will overflow from the microwell to the plastic dish bottom at this point. Islets are firmly attached to the ECM and will not float out of the microwell (Figure 2G–2I and 2N). The MatTek dish can hold 2 mL of media. The attached islets can be subjected to microscopy from day 4 to day 6.

**Day 6**

Islets attached firmly and further expanded their surfaces attached to the ECM. The area of attachment reaches 85%–90% of the hemispherical cross section area of an islet (Figure 2J–2L). It is noteworthy that the so-called “flattening” of isolated mouse islets refers only to the surface attached to the ECM-coated coverslip. The rest of the islet retains its spherical appearance even after 6 days of ex vivo culture. This is likely due to residual extracellular matrix (the peripheral capsule) surrounding an isolated mouse islet that maintains its spherical shape. Approximately 5% of attached islets can be fully flattened on a coated coverslip, likely due to incomplete peripheral capsule, and the islet periphery can reach single-cell-layer thickness (Figure 2O). The majority of attached islets with a hemispherical shape is suitable for live imaging using confocal or TRIF microscopy (Zhu et al., 2015; Trogden et al., 2021).

## Validation of protocol

This protocol or parts of it has been used and validated in the following research article(s):

- Trogden, K.P., Lee, J., Bracey, K.M., Ho, K.H., McKinney, H., Zhu, X., Arpag, G., Folland, T.G., Osipovich, A.B., Magnuson, M.A., Zanic, M., Gu, G., Holmes, W.R., Kaverina, I. (2021). Microtubules regulate pancreatic β-cell heterogeneity via spatiotemporal control of insulin secretion hot spots. *eLife* (Figure 2).
- Zhu, X., Hu, R., Brissova, M., Stein, R.W., Powers, A.C., Gu, G., Kaverina, I. (2015). Microtubules Negatively Regulate Insulin Secretion in Pancreatic β Cells. *Dev. Cell*. (Figure 2, panel C–F).

## Notes

1. A 10 μL tip may injure large islets during picking and transferring. We usually can obtain 200–300 islets from one mouse using this procedure. Do not use cell culture dish to incubate isolated islets to prevent their attachment to the dish.
2. The isolated islets are incubated in 3 mL of islet media in a 60 mm Petri dish overnight for recovery. The islet number should not exceed 100 per dish to minimize the formation of hypoxia core (necrosis due to insufficient O_2_) in larger islets.
3. The 10 mm microwell reduces the search time for an attached islet on a TIRF microscope and provides enough room for islets to attach to the glass coverslip instead of the edge of the microwell.
4. By choosing two islets with clear size difference, e.g., a mid-sized islet with a diameter of 100–120 μm and a small islet with a diameter of 60–80 μm, these two islets can be distinguished under the microscope without diamond pen marking on the coverslip.
5. Ideally, each 150 mm dish accommodates no more than six MatTek dishes to reduce the chance of knocking nearby MatTek dishes over while retrieving one.
